# Recombinant Human Adenovirus-*p53* Therapy for the Treatment of Oral Leukoplakia and Oral Squamous Cell Carcinoma: A Systematic Review

**DOI:** 10.3390/medicina57050438

**Published:** 2021-05-01

**Authors:** Jagadish Hosmani, Shazia Mushtaq, Shahabe Saquib Abullais, Hussain Mohammed Almubarak, Khalil Assiri, Luca Testarelli, Alessandro Mazzoni, Shankargouda Patil

**Affiliations:** 1 Department of Diagnostic Dental Sciences, College of Dentistry, King Khalid University, Abha 62529, Saudi Arabia; jhosmani@kku.edu.sa (J.H.); hualmubarak@kku.edu.sa (H.M.A.); alasery@kku.edu.sa (K.A.); 2 Dental Health Department, College of Applied Medical Sciences, King Saud University, Riyadh 11451, Saudi Arabia; smushtaqdr@gmail.com; 3 Periodontics and Community Dental Sciences, College of Dentistry, King Khalid University, Abha 62529, Saudi Arabia; sshahabe@kku.edu.sa; 4 Department of Oral and Maxillo Facial Sciences, Sapienza University of Rome, 00185 Rome, Italy; luca.testarelli@uniroma1.it (L.T.); alessandro.mazzoni@uniroma1.it (A.M.); 5 Department of Maxillofacial Surgery and Diagnostic Sciences, Division of oral Pathology, College of Dentistry, Jazan University, Jazan 45142, Saudi Arabia

**Keywords:** gene therapy, *rAD-p53* therapy, oral Leukoplakia, oral squamous cell carcinoma, gendicine

## Abstract

*Background and Objectives*: Oral cancer is the 6th most common cancer in the world and oral leukoplakia is an oral potentially malignant disorder that could develop into oral cancer. This systematic review focusses on randomized clinical trials for recombinant adenovirus *p-53* (*rAD-p53*) therapy for the treatment of oral leukoplakia and cancer. *Materials and Methods*: We searched for research articles on various databases such as Pubmed/Medline, Embase, CNKI (China National Knowledge Infra-structure), Springerlink, cochrane and Web of sciences from 2003 to 2020. MeSH (Medical Subject Headings) terms were used for the search. Inclusion criteria included original research, randomized clinical trials and articles only in English language. Exclusion criteria were any articles that were not research articles, not randomized trials, non-human studies, etc. The articles were further graded on the Jadad scale. *Results*: 578 articles were assessed from various databases; only 3 articles were found to be appropriate for this review. Thus, meta-analysis was not performed because of heterogeneity and lack of data. In the three studies, whether *rAD-p53* was used as a standalone therapy or with other therapies, there was a beneficial effect of the therapy. Furthermore, there were no serious adverse events and the only adverse events reported were fever, pain at the local injection site, flu-like symptoms and lowered WBC count. *Conclusions*: Thus, we can conclude that this therapy has a potential for beneficial therapeutic effects and further clinical trials with more patients need to be performed to get better understanding of the effect of *rAD-p53* therapy, which probably will pave the way to its approval in other parts of the world.

## 1. Introduction

Oral cancer is one the most prevalent cancers around the world (6th) and shows a much higher occurrence in Asia [1]. Oral leukoplakia (OLP), a white plaque formed on the oral mucosa, is considered an oral potentially malignant disorder (OPMD) [2,3,4]. The presence and grade of dysplasia are predictors of the risk of transformation of OLP to oral squamous cell carcinoma (OSCC) [4]. The number of cases where malignant transformation occurs varies in different parts of the world and is also dependent on tobacco consumption, some viral infections, and other exogenous factors [5].Anywhere between 1.5% to 34% of OLP transforms to OSCC, which is one the significant subset of head and neck squamous cell carcinomas (HNSCC) and accounts for almost 90% of all the oral cancers [6]. The primary aim of treatment for OLP is to prevent the progression to OSCC. The treatment for OSCC aims to eliminate cancer and avoid recurrence. If in later stages, I ensure the best quality of life possible without affecting the function of the organs [7]. The treatment that a patient receives for OLP depends on the spread of the lesion, type of OLP (classified after biopsy), and patient health [6]. The standard treatment includes surgery and topical/systemic medicines such as beta carotene, lycopene, vitamin A, bleomycin, etc. [8]. For OSCC, the treatment depends on the stage, primary site, location, proximity to lymph nodes and patient health [9]. The first line of treatment for early stage cancer has been surgery and sometimes, neck resections to prevent metastasis [9].

In the last 20 years or so, gene therapy has emerged as a treatment option for various diseases, including sarcomas and carcinomas [10]. Gene therapy involves manipulation of target DNA/RNA for the treatment of diseases, and various methods have been used, for example, viral-based delivery systems, cultured patient cells, etc. [10]. With the recent advances in the clustered regularly interspaced short palindromic repeats (CRISPR)/ CRISPR associated protein 9 (Cas9) tehnology, gene therapy is once more at the forefront of efforts to fight diseases [11].

Mutations in *P53*, a tumor suppressor gene, is a hallmark of human cancers and they have been observed in most cases of OLP and OSCC as well [12]. The inactivation of *p53* not only plays an important role in early tumor development, but also in proliferation of the tumor [13]. The mutant *p53* may function through various molecular mechanisms- it can bind to the DNA to alter gene expression. It can bind to transcription factors to improve or impede their functions or interact with proteins to change their function [14]. The restoration of *p53* function using the wild type *p53* with adenovirus as a delivery system, resulted in complete tumor regression in murine models and cell lines [15,16]. There have been clinical trials of potential Adenovirus-based therapy since 1990s [17,18]. Gendicine became the first gene therapy to be approved anywhere in the world in 2003, when China’s State Food and Drug Administration, approved the therapy developed by Shenzhen SiBiono GenTech. The recombinant Adenovirus *p53* gene (*rAD-p53*) therapy was approved for HNSCC [19]. Since then, 22 gene therapies have been approved till the end of 2019 [20]. Although there have been research on the efficacy of *rAD-p53* therapy for the treatment of HNSCC [21,22,23], the emphasis of this systematic review is on assessing the clinical benefits of using *rAD-p53* gene therapy for oral leukoplakia and OSCC as a stand-alone or in combination with other treatments. We examine the effectiveness of the drugs and speculate on their potential for OLP and OSCC.

## 2. Materials and Methods

The systematic review was registered with The International Prospective register for Systematic Reviews (PROSPERO) with ID number: CRD42020192065. The PICO framework was adhered to for this review: P- Population (Patients with oral leukoplakia or OSCC); I- Intervention (Adenovirus *p53* therapy + chemotherapy); C- Comparison between adenovirus *p53* therapy vs. traditional treatments; O- Outcomes (response rate, disease control rate, symptom improvement and adverse effects).

### 2.1. Search Method

A literature search was performed using Pubmed, Medline, Embase, SpringerLink, Ovid, Cochrane Library, Web of Science and CNKI (China National Knowledge Infrastructure). The MeSH terms used for the search were: “recombinant human adenovirus *p53* injection”, “*rAD-p53*”, “AD*p53* gene therapy”, “Gendicine”, “oral pre-malignant lesions”, “oral potentially malignant disorders”, and “oral leukoplakia”, “oral squamous cell carcinoma”, and/or “oral cancer”.

### 2.2. Inclusion and Exclusion Criteria

**Figure 1:**
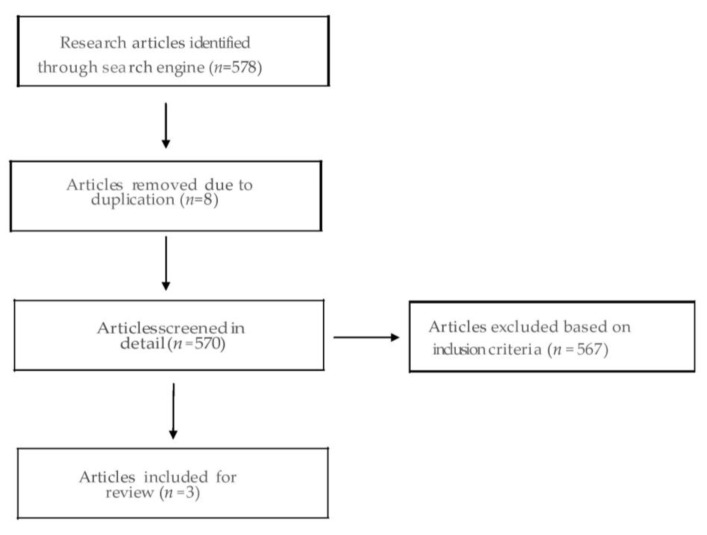
The flowchart showing database literature search and the method of shortlisting articles for review.

Inclusion criteria: (1) patients included in selected studies must have been diagnosed with OLP & OSCC clinically and histology; (2) study design must be clinical randomized controlled trial (RCT); (3) studies must have reported the outcome measures, which include response, (RR), disease control rate (DCR), symptom improvement (SI) and adverse effects (AEs); (4) Only English language studies. Exclusion criteria: (1) non-original articles, such as abstract, meeting record, editorial, review, book chapters and correspondence; (2) non-human studies; (3) in-vitro studies. The PRISMA flowchart is given in Figure 1.

### 2.3. Jadad Scoring

**Figure 2:**
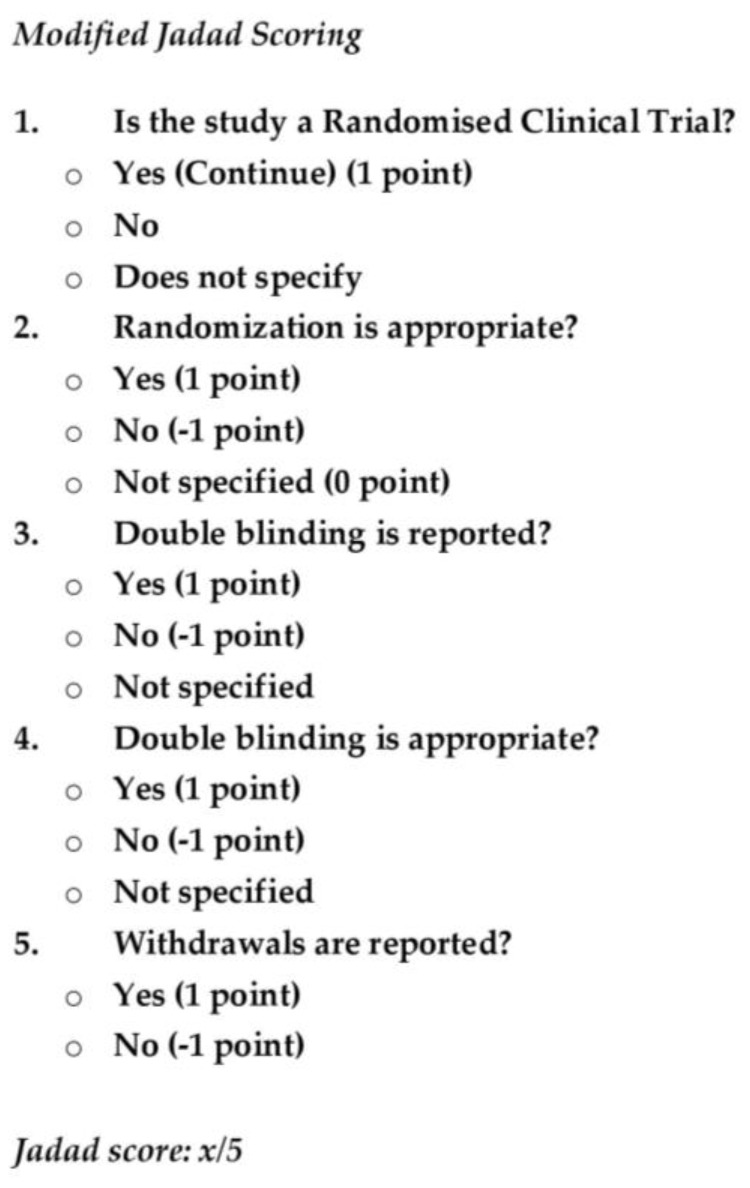
Modified Jadad questionnaire.

Jadad scoring system [24] was used to assess the methodological quality of the randomized clinical trials. The scoring questionnaire is presented in Figure 2.

#### Literature Search Summary

The search on various databases like pubmed/medline, CNKI, cochrane, embase with the MeSH terms resulted in a total of 578 articles. After all the inclusion and exclusion criteria were met, our list was reduced to 4 research articles. It was further reduced to 3 because one of the articles [25] was a report of the same subset of patients summarized in another article [26] (Table 1). We further used the JADAD scoring system to evaluate the methodological quality of the RCTs. The results are summarized in Table 2.

### 2.4. Outcome Measures

In the RCTs, the patients were followed up for 24–86 months after the treatment with rAD-53 and outcome measures such as response rate (RR), recurrence rate, symptom improvement (SI) were recorded. Any adverse events (AEs) associated with the treatment were also recorded, for example, fever, pain at injection site or any serious AEs (SAEs).

## 3. Results

### 3.1. Literature Search Summary

The search on various databases like pubmed/medline, CNKI, cochrane, embase with the MeSH terms resulted in a total of 578 articles. After all the inclusion and exclusion criteria were met, our list was reduced to 4 research articles. It was further reduced to 3 because one of the articles [25] was a report of the same subset of patients summarized in another article [26] (Table 1). We further used the JADAD scoring system to evaluate the methodological quality of the RCTs. The results are summarized in Table 2.

### 3.2. Synthesis of Evidence: Treatment, Follow-Up and the Outcome

The treatment and outcome measures for the 3 RCTs are summarized in Table 3. Li et al., 2009 [26] used *rAD-p53* therapy alone as a treatment option for 22 patients who had varying degrees of OLP in this phase I study. Gendicine was injected locally into the lesions. Out of the 22, 18 showed mucous membrane necrosis in the regions with lesions after treatment. 22.7% showed complete regression and showed no recurrence even after 24 months of treatment. 11/22 showed reduction in the size of the lesion area; 4 did not have any effect and 2 went on to develop cancer and had resection surgery. AEs reported were fever, pain in the injection area, flu-like symptoms after first injection and an increase in WBC count after therapy. Furthermore, immunohistochemical staining of biopsied tissues before and after treatment, saw differential and increased staining for *p53* (however *p* > 0.05) and *p21*^CIP/WAF^ (*p* < 0.01). The results are summarized in Table 3 *rAD-p53* clearly showed positive beneficial effects in the treatment of OLP.

Liu et al., 2013 [27] recruited a total of 107 patients with oral cancer (51 patients with tongue cancer and 56 with gingival cancer) primarily of stages II and III, and randomized them to either the experiment group (EG, 57 patients) or the control group (CG, 50 patients). The experimental group was injected with *rAD-p53* on the wound surface, after being treated with surgical resection and radiotherapy. The CG group only received the radiotherapy doses similar to EG group after the surgery, but no *rAD-p53* injection. The patients were all followed up for 36 months. In the EG group recurrence rate was 4/57, whereas in the CG group was 16/50, and this was statistically significant. The time of disease-free survival (DFS) was significantly higher for the EG group. The overall survival (OS) rate was 100% for EG group and 94% for CG group. There were no SAEs and the only AEs reported were fever and flu-like systems (Table 3). This Phase II clinical trial showed positive effects of *rAD-p53* therapy when combined with radiotherapy.

Li et al., 2014 [28] performed a phase III RCT (double-blinded and placebo-controlled) with 99 patients of oral carcinoma and used *rAD-p53* gene therapy in conjunction witFh chemotherapy. None of the patient had undergone any cancer treatment. The patients were diagnosed with either stage III or stage IV oral carcinoma and were randomly divided into three treatment groups: 35 patients in group I received intra-arterial infusion of *rAD-p53* and chemotherapy, 33 in group II received intra-arterial infusion of *rAD-p53* and placebo chemotherapy and 31 in group III received intra-arterial infusion of placebo *rAD-p53* and chemotherapy. There was a large variation in the follow up length from 3 months to 86 months because of advanced stages of carcinoma. Fifty-eight patients (16 in group I, 20 in Group II and 22 in group III) died during the follow up period. The primary lesions in 58 out of 92 patients showed response to therapy. The complete response plus the partial response rates were highest for group I (82%). Group II and Group III response rates were 53% and 54%, respectively. Group I showed the highest response rate for patients with stage IV carcinoma (Table 3). The rate of non-responders was also the lowest among the stage IV patients in group I. Patients with stage III carcinoma had higher survival rate in group I as compared to group III. Most common adverse events were flu-like symptoms and bone marrow suppression. However, gene therapy alone did not induce bone marrow suppression (group II), thus, it might have been a side-effect of chemotherapy. Immunohistochemistry was performed on the biopsy samples before and after treatment for *p53*, *bcl-2* (a protein downregulated by *p-53*) and Bax (a protein upregulated by *p-53*). The levels of these three proteins were same among the three groups. However, after treatment, the levels of *p53* expression was higher in group I. Higher Bax levels were observed in 85% of group I patients and 90% of group II patients, whereas in group III (no *rAD-p53* treatment), only 1 patient showed higher levels. Conversely, *bcl-2* expression was lower in 90% of patients in group I and 80% in group II. All the results and *p*-values are summarized in Table 3.

Thus, treatment with *rAD-p53* and chemotherapy together had better clinical outcomes as compared to either of them alone.

## 4. Discussion

### 4.1. Oral Cancer, Oral Leukoplakia and rAD-p53 Therapy: Summary of the Studies

Gene therapy for the treatment of various diseases, especially rare genetic disorders and solid carcinomas, is slowly gaining grounds as advances in genetic technology, such as, next-generation sequencing, better delivery vector designs, new gene editing technology such as CRISPR-CAS9, use of immune responses, make it safer and reliable [11,29,30,31]. Gendicine (commercial name for the *rAD-p53* therapy) became the first gene therapy across the world when it was approved by Chinese Food and Drug Administration [19]. *rAD-p53* gene therapy is either given as intra-arterial infusion or local wound injection and consists of introduction of an unmutated copy of *p53*, a known tumor suppressor gene, which induces apoptosis, necrosis and autophagy under cellular stress, thus leading to the death of tumor/cancer cells [13]. Since its approval, *rAD-p53* has been used as a therapy for other carcinomas such as nasopharyngeal cancer, non-small cell lung cancer, prostate cancer as well [18,22,32].

Oral cancer, a subtype of HNSCC, is one the most common cancers in the world and has been shown to be linked to lifestyle factors such as tobacco use, alcohol consumption and betelnut use [33]. The incidence of oral cancer across the world is highly dependent on these lifestyle factors. OSCC is the most common type of oral cancer and can affect the tongue, floor of the mouth, buccal cavity, etc. [7]. Oral leukoplakia, the potential pre-malignant lesions, lead to the development of OSCC in 1.5–34% of cases [6]. The traditional treatment methods of surgical resections, chemotherapy and radiotherapy have been successful, but in a limited way as the prognosis for survival at 5 years after diagnosis is still around only 50% [5]. Thus, the approval of Gendicine for the treatment of oral cancer and oral leukoplakia was a great news. However, since its approval, though it has been used for various types of cancer, their use for oral cancers and leukoplakia have been limited. We could only find three RCTs for *rAD-p53* use as a therapy for oral cancer or OLP. All the three studies showed clear beneficial effects of *rAD-p53* in the treatment of OLP or oral cancer when used alone or in combination with other traditional treatments. Li et al., used *rAD-p53* alone as a therapy in phase I study in 22 patients and saw complete regression in 22.7% and partial regression in 50% of the patients.

When *rAD-p53* was used along with surgery and radiotherapy, the survival was 100% till the follow up was done (36 months) [27]. Along with chemotherapy, it had significant beneficial effects for patients with stage III oral cancer [28]. Moreover, this therapy showed very few adverse events like flu-like symptoms, increase in WBC count and pain at injection site. There were no SAEs. The three studies were part of the clinical trials of the therapy. However, direct comparisons among the three studies is difficult to make on account of different study design, different phases of trial, and therapy was either given alone or in conjunction with other therapies, but no two studies had similar designs.

### 4.2. Mode of Action and Outcome of rAD-p53 Treatment

A conspicuous feature of *r*AD-*p53* is that it facilitates for seamless gene transfer as well as overt expression of *p53* protein in the nucleus of transfected cells. Regulating cell cycle progression, inducing programmed cell death, control on senescence of cells and autophagy are the important direct effects of *p53* protein [34].

Stalling cellular growth: *p53* protein regulates the checkpoints of different stages of cell cycle. *P53* enhances the manifestation of CDK inhibitor *p21*. This inhibitor attaches itself to cyclin E/cdk2 and cyclin A/cdk2 complexes and arrests the progression of cell cycle and replication of DNA. This step of inhibition permits for fixing damaged DNA. Management of S-phase checkpoints is attributed to *p53*-*p21* signaling axix. *p53* transcriptionally modulates manifestation of *Cdc25C*, *14-3-3σ*, *p21* and *GADD45* which has significant functional bearing on *G2/M* checkpoints [35].

Inciting programmed cell death: *p53* spells its cast vis-à-vis death receptor and mitochondrial pathways. Caspase cascade stimulation is ignited through the extrinsic, death receptor pathway. Apotosome formation takes place through the mitochondrial/intrinsic pathway, which leads to alteration in *Bcl-2* family proteins, leaning towards the proapoptotic members [36,37,38,39].

Controlling autophagy: This is an existence tactic and depends on the presence of *p53* at subcellular locations. Nuclear *p53* primarily impedes autophagy via transcription dependent mechanisms. Also, the *p53* invoked regulator of autophagy, DRAM, is crucial for programmed cell death. *r*AD-*p53* augments overexpression of *p53* thereby controlling the autophagy [40,41,42].

Instilling cell senescence: *p53* activated cellular senescence by transcriptional activation of target genes like *p21*, *PAI1*, and *PML* in riposte to oxidative stress or damage to DNA. *r*AD-*p53* enhances the expression of *p21* which induce cell senescence in malignancies [43].

*p53* executes through signalling and epigenetic tessellations. *r*AD-*p53* intratumor injections enable the transduced tumor cells to exhibit exalted levels of exogenetic *wt-p53*. The molecular mechanisms of *r*AD-*p53* are accredited primarily to binding of *p53* to the accompaniment/synergist elements of target genes and igniting cellular events that are related to tumor suppression. *p53* also regulates epigenetic changes occurring in cells [44]. Methylation, demethylation and post transcriptional modification are mediated by *p53* which lead to prevention of tumor formation [45].

### 4.3. rAD-p53 Gene Therapy in US

However, in spite of the success of this gene therapy in patients with HNSCC and also, for other solid tumors and carcinomas, the therapy has not been fully utilized because it has not been approved in other parts of the world for treatment. A therapy similar to Gendicine, Advexin, developed by Introgen was turned down by the FDA in September 2008. In fact, the first gene therapy to be approved in US was Kymriah in August 2017 for acute lymphoblastic leukemia (ALL).

Till now, there are more than 30 clinical trials using Gendicine as a therapy for various cancers only in China, where the therapy is approved [46]. It has mostly been used in conjunction with other standard intervention regimes. In fact, it was initially approved for use with chemotherapy or radiotherapy [46]. Since its approval, the mechanism of its action has been elucidated and the molecular pathways involved are much clear now [47,48,49]. Though it has been shown to be safe and had only minor adverse events, adenovirus as a vector can elicit host’s innate as well as adaptive immune responses. There have been development of safer adenovirus and other vectors to be used as a delivery system [50]. Furthermore, the therapy is not very expensive and pegged at around 390$ for one dose and a patient requires 5 doses [46,51]. Since it is not yet approved in most countries, we do not know how the health insurance might cover the costs. Further clinical studies determining the safety of the therapy are needed before it could be approved by the FDA which has stringent guidelines.

### 4.4. p53 as Biosignature for Gene Therapy

Tumor *p53* biosignature analysis may prognosticate *p53* gene therapy effectuality. Presence of normal *p53* gene sequences was indicator for favorable response to gene therapy in 116 recurrent HNSCC patients. In phase I and II trials, a statistically significant increase in tumor response was evident for patients with favorable *p53* efficacy profiles. In phase III trials too, statistically significant increases in time to progression and survival was noted in patients with favorable *p53* profiles [52]. *p53* mutation status can be utilized to predict the outcomes of radiotherapy and chemotherapy in HNSCC patients as per the observations chronicled by The Cancer Genome Atlas-based analysis of HNSCC patients [53].

## 5. Conclusions

*rAD-p53* gene therapy, approved by China in 2003, for HNSCC has been shown to be effective alone and in conjunction with standard regimes for oral cancer and OLP. However, lack of studies in populations other than Chinese, restricts its potential. More clinical studies, focusing on the safety of the therapy needs to be performed and as the mechanisms of its actions get elucidated further, we can hope that the therapy will be approved for treatment in other countries around the globe.

## Figures and Tables

**Table 1:** List of articles reviewed in this study and the study characteristics.

Author and Year	Study Type and Design	Sample Population; Age Range	*rAD-p53* Alone or with Other
Li et al., 2009 [24]	Oral lichen planus (OLP), RCT, Phase I, Blinding not defined	22; 35–74; Male (M) and Female (F)	*rAD-p53* alone
Liu et al., 2013 [25]	Oral cancer, RCT, Phase II	107; 27–80; M and F	radical resection followed by radiotherapy and *rAD-p53*
Li et al., 2014 [26]	Oral cancer; RCT, double-blinded, Phase III	99; mean age around 57; M and F	*rAD-p53* and chemotherapy

**Table 2:** The Jadad scores of the three articles used for this systematic review.

Article and Year Published	Jadad Score
1. Li et al., 2009 [24]	2/5 (no blinding)
2. Liu et al., 2013 [25]	4/5 (unmasked)
3. Li et al., 2014 [26]	5/5 (double-blind)

**Table 3:** Summary of results from the three RCTs.

Article and Year	Primary Outcomes (%)	*p*-Value	Adverse Events	*p*-Value	IHC Pre- vs. Post Treatment (%)
Li et al., 2009 [24]	Complete regression 22.7 Partial regression 50 No effect 18.2 Progress to cancer 9	NA	Transient fever 31.8 Injection site pain 22.7 Flu-like symptoms 9.1 Increase in WBC 18.2	NA	*p-53* low or negative vs. high in all samples *p-21*^CIP/WAF^ 22.7 vs. 86.4 *Bcl-2* 54.5 vs. 18.2
Liu et al., 2013 [25]	TC recurrence: EG 7.4 CG 33.3 GC recurrence: EG 6.7 CG 30.8 3-year OS: EG 100 CG 94 3-year DFS EG 93 CG 68	0.0326 0.0332 0.0586 0.0002	Transient Fever: EG 84 CG 12 Flu-like symtomps EG 64 CG 4 Oral Membrane EG 24.6 Burn and pain CG 30	<0.0001 <0.0001 0.6634	NA
Li et al., 2014 [26]	CR: G I 48.5 GII 16.7 GIII 17.2 PR: GI 33.3 GII 36.7 G III 34.5 SD or PD: GI 18.2 GII 46.7 GIII 48.3 Recurrence: GI 9.1 GII 20.0 GIII 24.1	0.006 0.961 0.020 0.267	Flu-like symptoms GI 81.8 GII 76.7 GIII 55.2 Bone Marrow Suppression GI 36.4 GII 0.0 GIII 37.89	0.051	Bax Increase GI 84.8 GII 90.0 GIII 3.4 Bcl-2 decrease GI 90.0 GII 80.0 GIII 27.6

NA, Not Applicable; IHC, Immunohistochemistry.TC, Tongue Cancer; GC, Gingival Cancer; OS, Overall Survival; DFS, Disease Free Survival; EG, Experimental Group receiving *rAD-p53*+ radiotherapy; CG, Control Group receiving only radiotherapy. G I, Group I, *rAD-p53* + chemotherapy; G II, Group II, *rAD-p53* + placebo chemotherapy; G III, placebo *rAD-p53* + chemotherapyCR, Complete Response; PR, Partial Response; PD, Progressive Disease; SD, Stagnant Disease.

## Data Availability

Not applicable.
